# Free DNA and Metagenomics Analyses: Evaluation of Free DNA Inactivation Protocols for Shotgun Metagenomics Analysis of Human Biological Matrices

**DOI:** 10.3389/fmicb.2021.749373

**Published:** 2021-10-06

**Authors:** Leonardo Mancabelli, Christian Milani, Rosaria Anzalone, Giulia Alessandri, Gabriele Andrea Lugli, Chiara Tarracchini, Federico Fontana, Francesca Turroni, Marco Ventura

**Affiliations:** ^1^Laboratory of Probiogenomics, Department of Chemistry, Life Sciences and Environmental Sustainability, University of Parma, Parma, Italy; ^2^Interdepartmental Research Centre “Microbiome Research Hub”, University of Parma, Parma, Italy; ^3^GenProbio srl, Parma, Italy

**Keywords:** propidium monoazide, free DNA, shotgun metagenomics, human microbiome, real-time PCR

## Abstract

Culture-independent approaches now represent the gold standard for the investigation of both environmental and host-associated complex microbial communities. Nevertheless, despite the great advantages offered by these novel methodologies based on the use of next-generation DNA sequencing approaches, a number of bias sources have been identified. Among the latter, free DNA contained in biological matrices is one of the main sources of inaccuracy in reconstructing the resident microbial population of viable cells. For this reason, the photoreactive DNA-binding dye propidium monoazide (PMAxx^™^) has been developed by improving standard PMA. This compound binds and inactivates free DNA, thus preventing its amplification and sequencing. While the performances of PMA have been previously investigated, the efficiency with PMAxx^™^ has been tested mainly for amplicon-based profiling approaches on a limited number of biological matrices. In this study, we validated the performance of PMAxx^™^ for shotgun metagenomics approaches employing various human-associated matrices. Notably, results revealed that the effectiveness of PMAxx^™^ in inactivating free DNA of prokaryotes and eukaryotes tends to vary significantly based on the biological matrices analyzed.

## Introduction

The advent of modern culture-independent bacterial DNA sequencing technologies allows to achieve an in-depth characterization of the microbial communities inhabiting the human and animal bodies as well as the microbial consortia residing in other environments (Browne et al., 2016; Milani et al., 2017). These innovative metagenomics approaches, such as 16S rRNA gene sequencing or shotgun metagenomics, have found great relevance in the human field, revealing a close mutual interaction between the microorganisms and their hosts (Milani et al., 2017; Lebeer and Spacova, 2019; Zheng et al., 2020). Moreover, the microorganisms colonizing the human body, defined as microbiota, seem to play a key role in several physiological functions of the host, such as metabolism, pathogen exclusions, and development of the host immune system (Belkaid and Hand, 2014; Ubeda et al., 2017; Fan and Pedersen, 2021). The use of culture-independent methodologies has several advantages, such as the possibility to rapidly identify bacterial communities that are hardly observed with culture-based studies, the opportunity to freeze samples, high specificity, and efficiency in the identification of bacterial phylogeny and taxonomy, reproducible and technically easy procedure, accessible bioinformatic pipelines, and low cost (Cocolin et al., 2013; Zapka et al., 2017; Mancabelli et al., 2020). However, culture-independent methodologies can introduce inherent biases, including the risk of bacterial contamination before and during sample storage, inadequate or preferential disruption of specific bacterial cells, the use of different DNA extraction methods and methodological analyses, and the inability to exclude non-viable DNA (Leff et al., 1995; Schneegurt et al., 2003; Rogers et al., 2013; Young et al., 2017). Specifically, the persistence of non-viable DNA could involve imprecise cataloging of the bacterial communities of a specific biological sample and an overestimation of the number of bacterial species richness and complexity (Brooks et al., 2015; Young et al., 2017). In this context, an important criterion used to distinguish viable cells vs. damaged cells is represented by the integrity of the cell membrane (Rudi et al., 2005; Nocker et al., 2006). In fact, viable bacteria with intact cell membranes could be distinguished by the use of specific DNA-binding dyes that easily penetrate dead or membrane-compromised cells. The most common membrane-impermeant dye is propidium iodide (PI), which is largely applied to distinguished live from dead bacterial cells using microscopic and flow cytometry approaches (Nocker et al., 2006). In the last decade, real-time PCR approaches combined with DNA-intercalating dye treatment allowed to readily discriminate viable microorganisms in complex bacterial populations (Rudi et al., 2005). A recent study reported that the largely used DNA-intercalating dye, i.e., ethidium monoazide, also may penetrate viable cells inducing partial DNA loss (Nocker et al., 2006, 2007), suggesting the need of chemical alternative dyes such as propidium monoazide (PMA). PMA is a DNA chelating compound that cannot translocate across a viable cellular membrane, allowing to discriminate viable and non-viable bacterial cells with high efficiency (Nocker et al., 2007, 2010). Several metagenomic studies reported the effectiveness of PMA to selectively remove DNA from prokaryotes and eukaryotes dead cells during the early stages of sequencing processes. In particular, studies based on 16S rRNA gene sequencing have shown significant differences in bacterial community composition and α-diversity between treated and untreated samples (Nguyen et al., 2016; Li et al., 2017; Young et al., 2017; Mo et al., 2019; Stinson et al., 2019; Marotz et al., 2021), highlighting the accuracy of PMA. Furthermore, PMA has also been applied to samples involved in shotgun metagenomics studies (Marotz et al., 2018, 2021), thus revealing the capability of PMA to remove free DNA from biological samples. However, the majority of these validation studies focused on a limited range of biological matrices harboring a simple community of microorganisms, such as human saliva (Marotz et al., 2018, 2021), infant meconium (Young et al., 2017; Stinson et al., 2019), and water (Li et al., 2017). In this study, we performed a comprehensive evaluation of the efficiency of the new and improved version of PMA, i.e., PMAxx^™^, on synthetic and complex microbial communities when the corresponding extracted DNA is submitted to both amplicon-based and shotgun-based approaches.

## Materials and Methods

### Evaluation of Microbial Cell Load

Cells of *Bifidobacterium bifidum* PRL2010 were recovered from an overnight culture, and turbidity was measured at 600nm using a biophotometer (Eppendorf). A growth tube containing 6ml of MRS was inoculated with active viable bacterial cells diluted to an OD_600nm_ of ~1.0, obtaining a final inoculum with an OD_600nm_ of ~0.1. PMAxx^™^ (Biotium Inc., CA, United States) was added at a concentration of 100μM. Cultures were grown in biologically independent triplicates, and the resulting growth datasets were expressed as the means from these replicates. Moreover, positive growth controls without PMAxx^™^ were performed. Cultures were incubated under anaerobic conditions at 37°C for 24h. Cell growth was monitored using a Thoma cell counting chamber according to the producer’s instructions (Herka).

### Real-Time PCR

The efficiency of PMAxx^™^ was evaluated through quantitative real-time PCR (RT-PCR). In detail, we tested the efficiency of PMAxx^™^ on free bacterial DNA of *B. bifidum* PRL2010, *Lactobacillus crispatus* PRL2021, and *Escherichia coli* Nissle 1917 bacterial strains and on free eukaryotic DNA of *Saccharomyces cerevisiae* ATCC18824, separately. The DNA of each strain was extracted and diluted at concentration of 1ng, 10ng, and 100ng. Each concentration was treated with PMAxx^™^ at 50μM and 100μM. The presence of free DNA of the strains was evaluated using quantitative real-time PCR (qRT-PCR). The primer pair used in this study is Probio_uni/Probio_rev (5′-CCTACGGGRSGCAGCAG-3′/5′-ATTACCGCGGCTGCT-3′) (Milani et al., 2013) and BITS/B58S3 (5′-ACCTGCGGARGGATCA-3′/5′-GAGATCCRTTGYTRAAAGTT-3′) (Bokulich and Mills, 2013) for bacterial and eukaryotic DNA amplification, respectively. RT-PCR was performed using SoFast EvaGreen Supermix (Bio-Rad) on a CFX96 system (BioRad, CA, United States) following previously described protocols (Milani et al., 2015). Each PCR reaction mix contained the following: 12.5μl 2x SYBR SuperMix Green (BioRad, CA, United States), 5μl of DNA at the concentration of 10ng/μl, each of the forward and reverse primers at 0.5μM, and nuclease-free water was added to obtain a final volume of 20μl.

### Heat Treatment

Fresh cultures of *B. bifidum* PRL2010, *L. crispatus* PRL2021, and *E. coli* Nissle 1917 were devitalized using heat. In detail, 1ml aliquot of each culture was exposed to 95°C for 5min in a heating block.

### Bacterial Mock Community

The cultures of 13 different bacteria strains were grown separately on an acknowledged species-specific medium (Supplementary Table S1) and supplemented with 0.05% (wt/vol) L-cysteine hydrochloride, followed by incubation in an anaerobic atmosphere (2.99% H2, 17.01% CO_2_, and 80% N2) in a Concept 400 chamber (Ruskin) at 37°C until they reached late log phase. Subsequently, the optical density at 600nm of each culture was measured, and cells were diluted to obtain an OD600nm=1. In detail, a total of three bacterial mock communities were obtained combining total equal volumes (20μl) of each selected bacterial viable cells diluted an OD600nm=1. Moreover, chromosomal DNA of each strain added to the bacterial mock communities as free DNA was extracted as previously described (Milani et al., 2015). More details regarding the composition of the mocks are shown in Table 1.

**Table 1:** Bacterial composition of mock communities.

	Viable cells	Free DNA
Mock_1	*Lactobacillus crispatus*	*Bifidobacterium bifidum*
	*Lactobacillus helveticus*	
	*Lactococcus lactis*	
	*Staphylococcus epidermidis*	
	*Collinsella aerofaciens*	
	*Eryspelatoclostridium ramosum*	
	*Bacteroides dorei*	
	*Acidaminococcus intestinii*	
	*Faecalicoccus pleomorphus*	
	*Bacteroides vulgatus*	
Mock_2	*Bifidobacterium adolescentis*	*Bifidobacterium bifidum*
	*Bifidobacterium breve*	
	*Lactobacillus helveticus*	
	*Lactococcus lactis*	
	*Collinsella aerofaciens*	
	*Eryspelatoclostridium ramosum*	
	*Bacteroides dorei*	
	*Acidaminococcus intestinii*	
	*Faecalicoccus pleomorphus*	
	*Bacteroides vulgatus*	
Mock_3	*Lactobacillus helveticus*	*Bifidobacterium adolescentis*
	*Lactococcus lactis*	*Bifidobacterium breve*
	*Collinsella aerofaciens*	*Staphylococcus epidermidis*
		*Eryspelatoclostridium ramosum*
		*Acidaminococcus intestinii*
		*Faecalicoccus pleomorphus*

### PMA Treatment of Mock Community and Bacterial DNA Extraction

PMAxx^™^ (Biotium Inc., CA, United States) was mixed with each mock to the final concentration of 75μM, 100μM, and 150μM. Afterward, the samples were placed on ice for 30min, in the dark with intermittent mixing. Subsequently, samples were exposed to blue LED light at 464nm at 30-s intervals for a total of 2min (Young et al., 2017). Therefore, samples were centrifuged at 10,000×*g* for 5min (Young et al., 2017). The supernatant was discarded, and the remaining DNA was extracted using GenElute^™^ Bacterial Genomic DNA Kits (Sigma-Aldrich) according to the manufacturer’s instructions.

### PMA Treatment of Biological Samples and Bacterial DNA Extraction

Human samples of saliva, feces, urine, and a vaginal swab were added with free DNA of the bacterial species *Gordonia amicalis* DSM44461 (50ng in 1ml of each biological sample) and mixed with PMAxx^™^ and treated as described above for mock communities (Supplementary Table S2). In addition, saliva, feces, urine, and vaginal samples were subjected to DNA extraction using QIAmp DNA Mini Kit, QIAmp DNA stool Mini Kit, i-genomic Urine DNA Extraction Mini Kit, and ZymoBIOMICS DNA miniprep kit, respectively, following the manufacturer’s instructions.

### Shallow-Shotgun Metagenomics

The extracted DNA was prepared using the Illumina Nextera XT DNA library preparation kit. The DNA samples were enzymatically fragmented for a short time, barcoded, and purified involving magnetic beads (AmpliClean^™^ Cleanup kit). Previously samples were quantified using the fluorometric Qubit quantification system (Life Technologies, United States). The samples were then loaded on a 2,200 TapeStation instrument (Agilent Technologies, United States) and normalized to 4nM. DNA sequencing was performed paired-end using an Illumina MiSeq sequencer with flow cell v3 600 cycles (Illumina Inc., San Diego, United States). DNA sequencing generated fastq format files for each sample. The fastq files obtained were filtered for quality (>20) and length (>80bp) of the reads (Milani et al., 2018, 2021). The filtered data were then used for mapping the analyzed samples against the reference genome of species added as free DNA to mock communities and real biological samples using the software package BWA (Burrows-Wheeler Aligner) (Li and Durbin, 2010).

### Data Deposition

Raw sequences of the shallow-shotgun metagenomics experiments are accessible through SRA study accession numbers PRJNA750324.

## Results and Discussion

### Evaluation of PMAxx^™^ Accuracy on Viable Bacterial Cells

**Figure 1:**
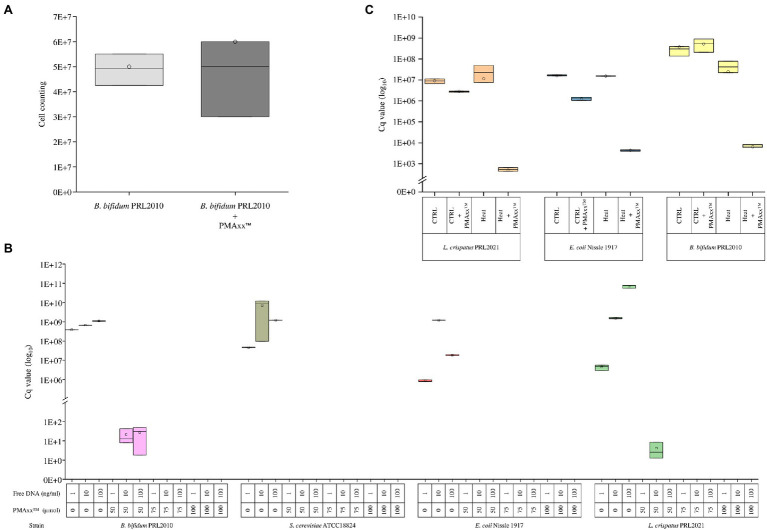
Evaluation of the PMAxx^™^ efficacy on simple bacterial communities. Panel **A** shows the bacterial load of *B. bifidum* PRL2010 cells treated and no-treated with PMAxx^™^. In the whiskers plot, the y-axis indicates cell counting. The boxes are determined by the 25th and 75th percentiles. The whiskers are determined by the maximum and minimum values and correspond to the boxes’ extreme values. The line in the boxes represented the average, while the circle represents the median. Panel **B** reveals the qPCR results based on DNA extracted from *B. bifidum* PRL2010, *L. crispatus* PRL2021, *E. coli* Nissle 1917, and *S. cerevisiae* ATCC18824, respectively. Each strain was evaluated with a different DNA volume and with or without PMAxx^™^ treatment. In the whiskers plot, the y-axis indicates the Cq value. The boxes are determined by the 25th and 75th percentiles. The whiskers are determined by the maximum and minimum values and correspond to the boxes’ extreme values. The line in the boxes represented the average, while the circle represents the median. Panel **C** reports the qPCR results performed on *B. bifidum* PRL2010, *L. crispatus* PRL2021, and *E. coli* Nissle 1917, respectively. Each strain was treated and not treated with heat and treated and not treated with PMAxx^™^. In the whiskers plot, the y-axis indicates the Cq value. The boxes are determined by the 25th and 75th percentiles. The whiskers are determined by the maximum and minimum values and correspond to the boxes’ extreme values. The line in the boxes represented the average, while the circle represents the median.

PMAxx^™^ is a photoreactive DNA-binding dye used for quantitative PCR (qPCR) derived from chemical improvement of PMA.^1^ When exposed to visible light, PMAxx^™^ binds to DNA with high affinity through covalent bonds and inhibits processes such as amplification by PCR (Nocker et al., 2006). Moreover, PMAxx dye is a cell membrane-impermeant, which binds only to non-viable bacterial cells with a compromised membrane. In this context, the bacterial cell integrity was measured following the PMAxx^™^ treatment on different bacterial cultures. In detail, we have grown cells of the strain *B. bifidum* PRL2010 on MRS broth in biologically independent triplicates, which were subsequently treated with PMAxx^™^. Moreover, an additional control replicate not treated with PMAxx^™^ was performed. The bacterial load (Figure 1A) did not reveal any significant differences (*p* value >0.05) between cells treated with PMAxx^™^ and controls, confirming that PMAxx^™^ does not damage the cell structure of viable cells.

Furthermore, in order to evaluate the efficiency of PMAxx^™^ in the process of free DNA inactivation, DNA extracted from three microorganisms, i.e., *B. bifidum* PRL2010, *L. crispatus* PRL2021, and *E. coli* Nissle 1917, as well as DNA of *Saccharomyces cerevisiae* ATCC18824, was evaluated separately through quantitative real-time PCR (qPCR). For each strain, 1ng, 10ng, and 100ng diluted in 1ml of water were evaluated by qPCR without treatment or with the addition of 50, 75, or 100μmol of PMAxx^™^ (Figure 1B). The analysis confirmed the accuracy of PMAxx^™^ in binding free DNA and inhibiting the amplification by PCR. In detail, PMAxx^™^ concentration of 75μmol and 100μmol showed the total inhibition of PCR amplification for all samples tested. In contrast, the PMAxx^™^ at concentration of 50μmol did not inhibit the amplification in all conditions tested, such as for *B. bifidum* PRL2010 and *L. crispatus* PRL2021 samples (Figure 1B). These results revealed that the concentration of 75μmol pf PMAxx^™^ was adequate to deplete free DNA and could represent an efficacy cutoff.

### Ability of PMAxx^™^ to Inhibit DNA Released by Dead Cells

The ability of PMAxx^™^ to remove free DNA released by dead bacterial cells was evaluated by heat-killing fresh cultures of *B. bifidum* PRL2010, *L. crispatus* PRL2021, and *E. coli* Nissle 1917, separately. In detail, the amount of free DNA was evaluated for both viable control cells and heat-treated cells of each strain through qPCR. The analysis confirmed that PMAxx^™^ is able to inactivate the free DNA released by cells lysed by heat treatment, as demonstrated by comparable DNA amount detected in viable control cells (*p* value >0.05) (Figure 1C). As expected, these results confirmed the high efficiency of PMAxx^™^ in binding exclusively with free DNA (Li et al., 2017; Marotz et al., 2021; Wang et al., 2021), highlighting a significant decrease in the amount of DNA only in samples treated with heat and PMA (Figure 1C).

### Validation of Free DNA Removal With Mock Communities

**Figure 2:**
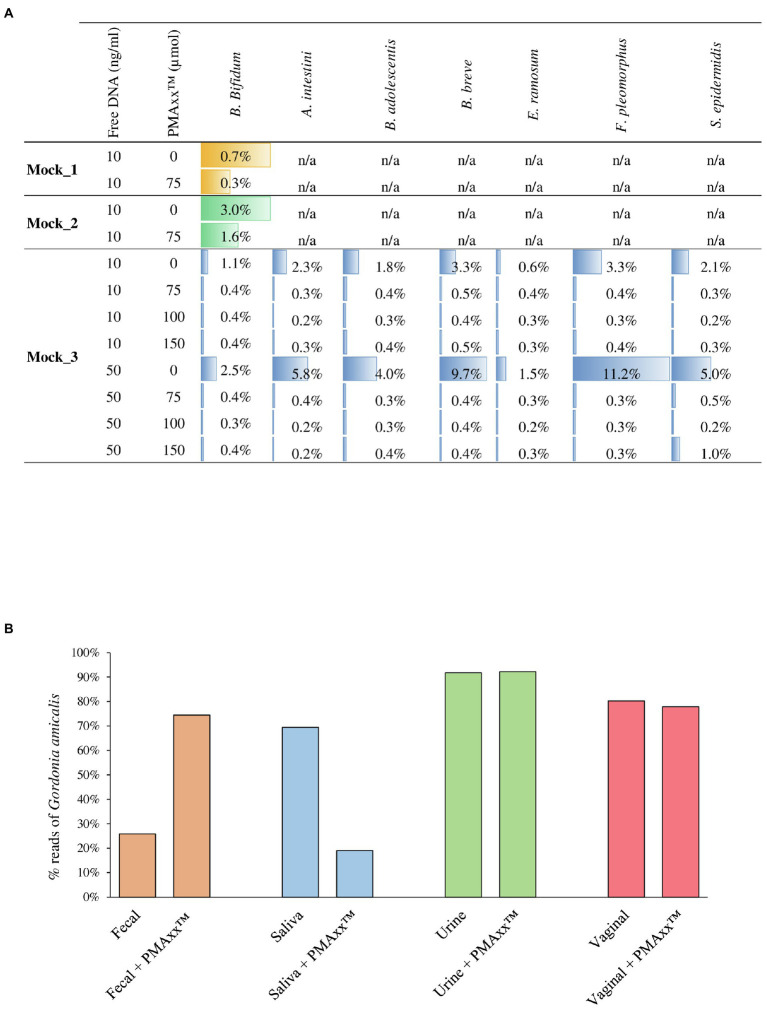
Assessment of the PMAxx^™^ efficiency on artificial microbial communities and biological samples. Panel **A** shows for each mock community performed, the percentage of sequences aligned to the specific added free DNA. Panel **B** reports the percentage of *G. amicalis* DSM44461 free DNA in each biological sample, i.e., fecal, saliva, urine, and vaginal, treated or no-treated with PMAxx^™^.

In order to verify the efficiency of PMAxx^™^ treatment on complex bacterial communities, 13 different bacterial cultures were grown separately (Supplementary Table S1) and were subsequently used to constitute three bacterial artificial communities named Mock_1, Mock_2, and Mock_3 (Table 1). The bacterial species used to assemble the mock communities were selected as representative of the human microbiota. Moreover, chromosomal DNA was extracted from seven strains and added to each mock community as free DNA, as reported in Table 1. Therefore, each mock was treated with PMAxx^™^ and submitted to DNA extraction, followed by shotgun metagenomics sequencing (Supplementary Table S2). In detail, 1ml of Mock_1 and Mock_2 were treated with 75μmol of PMAxx^™^. Analysis of the datasets obtained from Illumina sequencing revealed that the percentage of metagenomic sequences corresponding to the added free DNA of *Bifidobacterium bifidum* LMG11041 dropped by 58 and 46% in Mock_1 and Mock_2, respectively (Figure 2A).

Moreover, in order to test the performances of different concentrations of PMAxx^™^, 1ml of Mock_3 was processed, respectively, with 75, 100, and 150μmol of PMAxx^™^ and then submitted to DNA extraction and subsequently to shotgun sequencing (Supplementary Table S2). Remarkably, the free DNA retrieved from seven different bacterial species was successfully inactivated in all cases disregarding the amount of free DNA added but with an efficiency proportional to the amount of PMAxx^™^ applied (Figure 2A). Notably, despite the different amounts of free DNA and PMAxx^™^ used, in all cases, an average of 0.36±1.13% of the sequencing reads still correspond to a bacterial species added to the mock as free DNA (Figure 2A). Thus, underlining that persistence of a small amount of active free DNA should always be expected.

### Validation of Free DNA Removal Using Biological Samples

The effectiveness of 75μmol of PMAxx^™^ for the inactivation of free DNA was also tested using real biological samples obtained from a healthy individual. In detail, a sample of saliva, feces, urine, and a vaginal swab were collected and added with free DNA of the bacterial species *Gordonia amicalis* DSM44461 (50ng in 1ml of each biological sample; Supplementary Table S2). Before adding free DNA, the presence of species belonging to *Gordonia* genus in each sample was verified by qPCR using G268F/G1096R primer pairs (Shen and Young, 2005), highlighting the absence of these bacteria in the assayed samples. As previously reported in the literature, treating the saliva sample with PMAxx^™^ resulted in 73% reduction in the sequencing reads corresponding to the added free DNA (Marotz et al., 2018, 2021; Wang et al., 2021; Figure 2B). In contrast, the percentage of sequencing reads corresponding to *G. amicalis* DSM44461 observed in the fecal sample increases after treatment with PMAxx^™^. A possible explanation for this finding is that the fecal sample is rich in free DNA, both eukaryotic and prokaryotic, due to digestion of food components and dead bacterial cells detaching from the intestinal mucosa. Thus, a generalized reduction of this high amount of free DNA may increase the relative abundance of the added free DNA of *G. amicalis* DSM44461 although the absolute abundance of the latter has been lowered (Figure 2B).

Peculiar results were also observed for the urine and vaginal swab samples. In these cases, the artificially added free DNA was not affected by treatment with PMAxx^™^ (Figure 2B). Notably, these unexpected results suggest that the chemical composition of the biological sample may influence the PMAxx^™^ activity. These preliminary results remark the need for extensive and specific validation of the use of PMAxx^™^ for free DNA inactivation in different biological samples since the results may vary extensively.

## Conclusion

This study aimed at investigating the performance of PMAxx^™^, a photoreactive DNA-binding dye which is a modified PMA with improved performances in inactivating free DNA by binding covalently when exposed to visible light (Nocker et al., 2006). While a range of studies evaluated performances of PMA, very little is known about the efficacy of PMAxx^™^ in reducing/abolishing the presence of free DNA in biological matrices. Through the involvement of artificial microbial communities and biological samples, we validated the use of PMAxx^™^ following the main methodologies previously tested with standard PMA. Intriguingly, the comparison of the performances retrieved from different biological matrices revealed matrix-dependent performance of PMAxx^™^. These data underline the need for matrix-specific validation of PMAxx^™^ performances and in-depth investigation of the chemical/physical causes of the observed reduction in DNA-binding and inactivation.

## Data Availability Statement

The datasets presented in this study can be found in online repositories. The names of the repository/repositories and accession number(s) can be found in the article/Supplementary Material.

## Author Contributions

LM processed the metagenomic data, conducted the analyses, and wrote the manuscript. CM and FT participated in the design of the study. CM contributed to the manuscript preparation. RA performed *in vitro* analyses. FF contributed to the statistical analyses. GL and CT contributed to the metagenomic analyses. MV conceived the study, participated in its design and coordination, and contributed to the manuscript preparation. All authors have read and approved the final manuscript.

## Funding

This study was supported by “Programma Operativo Nazionale Ricerca e Innovazione” 2014–2020 (PON “R&I” 2014-2020) (project ARS01_00530) and by University of Parma (project COVIDbiome). FT was supported by PROGETTO Ricerca Finalizzata, Ministero della Salute (RF GR-2018-12365988).

Part of this research is conducted using the High Performance Computing (HPC) facility of the University of Parma.

## Conflict of Interest

RA was employed by the company GenProbio srl.

The remaining authors declare that the research was conducted in the absence of any commercial or financial relationships that could be construed as a potential conflict of interest.

## Publisher’s Note

All claims expressed in this article are solely those of the authors and do not necessarily represent those of their affiliated organizations, or those of the publisher, the editors and the reviewers. Any product that may be evaluated in this article, or claim that may be made by its manufacturer, is not guaranteed or endorsed by the publisher.

## Footnotes

^1^ https://biotium.com/product/pmaxx-20-mm-in-h2o/
